# Supplementary material for: Treatment Pattern, Financial Burden, and Outcomes in Elderly Patients with Acute Myeloid Leukemia in Korea: A Nationwide Cohort Study
Source: Int J Environ Res Public Health. 2022 Feb 17;19(4):2317. doi: 10.3390/ijerph19042317 (PMC8872510; doi:10.3390/ijerph19042317)
Supplement: Supplementary file 1 [file ijerph-19-02317-s001.zip › ijerph-1529183-supplementary.pdf]

Supplementary Table S1. Diagnostic code of acute myeloid leukemia according to the International Statistical Classification of Diseases tenth revision (ICD-10).

| ICD-10 code | Diagnosis |
|---|---|
| C92 | Myeloid leukemia |
| C93 | Monocytic leukemia |
| C94 | Other leukemias of specified cell type |
| C95 | Leukemia of unspecified cell type |

* Excluding the diagnostic codes below
C92.1: chronic myeloid leukemia
C92.2: subacute myeloid leukemia
C92.3: myeloid sarcoma
C93.1: chronic monocytic leukemia
C94.4: acute panmyelosis
C94.5: acute myelofibrosis
C94.6: myelodysplasic and myeloproliferative disease, not classified
C95.1: chronic leukemia of unspecified cell type

Supplementary Table S2. Code of chemotherapeutics agents according to the Anatomic Therapeutic Chemical Classification (ATC)

| ATC code | Chemotherapeutics |
|---|---|
| 139601BIJ, 139602BIJ, 139603BIJ, 139604BIJ, 139605BIJ, 139606BIJ, 139607BIJ, 139608BIJ, 139609BIJ, 139630BIJ, 139631BIJ, 139632BIJ, 139633BIJ, 139634BIJ, 139635BIJ, 139636BIJ, 139637BIJ, 139638BIJ, 139639BIJ, 139640BIJ | cytarabine |
| 248001BIJ, 248002BIJ, 248003BIJ, 248030BIJ, 248031BIJ, 248032BIJ | vincristine sulfate |
| 192101ATB, 192101BIJ, 192102BIJ, 192103BIJ, 92104BIJ, 192105BIJ, 192106BIJ, 192107ATB, 192107BIJ, 92108BIJ, 192109BIJ, 192110BIJ, 192111BIJ, 192112BIJ, 192132BIJ, 192134BIJ, 192136BIJ, 192138BIJ, 192139BIJ, 192140BIJ, 192141BIJ, 192142BIJ, 192143BIJ, 192144BIJ, 192145BIJ, 192146BIJ | methotrexate |
| 190601ATB | mercaptopurine hydrate |
| 181401BIJ, 181402BIJ, 181403BIJ | L-asparaginase |
| 139001ATB, 139002BIJ, 139003BIJ, 139004BIJ, 139005BIJ | cyclophosphamide |
| 196502BIJ, 196501BIJ, 196530BIJ, 196531BIJ | mitoxantrone |
| 172001ACH, 172002ACH | hydroxyurea |
| 237901ATB | thioguanine |
| 243001ACS, 243002CCM, 243002COM, 243003CCM, 243003COM, 243004CCM, 243004CLQ, 243004COM, 243005CCM, 243009CLQ | tretinoin |
| 495602BIJ, 495601BIJ | decitabine |
| 484301BIJ, 484302BIJ | azacitidine |
| 149430BIJ, 149431BIJ, 149432BIJ, 149433BIJ, 149434BIJ, 149435BIJ | doxorubicin hydrochloride |
| 140601BIJ | daunorubicin hydrochloride |
| 173001ACH, 173002ACH, 173002BIJ | idarubicin |
